# Dynamic cerebral autoregulation in anterior and posterior cerebral circulation during cold pressor test

**DOI:** 10.1186/s12576-020-00732-7

**Published:** 2020-01-29

**Authors:** Takuro Washio, Hironori Watanabe, Shigehiko Ogoh

**Affiliations:** 1 Department of Biomedical Engineering, Toyo University, 2100 Kujirai, Kawagoe-shi, Saitama, 350-8585 Japan; 2 Research Fellow of Japan Society for the Promotion of Science, Tokyo, Japan

**Keywords:** Posterior cerebral artery, Middle cerebral artery, High blood pressure, Sympathoactivation

## Abstract

We hypothesized that cerebral blood flow (CBF) regulation in the posterior circulation differs from that of the anterior circulation during a cold pressor test (CPT) and is accompanied by elevations in arterial blood pressure (ABP) and sympathetic nervous activity (SNA). To test this, dynamic cerebral autoregulation (dCA) in the middle and posterior cerebral arteries (MCA and PCA) were measured at three different conditions: control, early phase of the CPT, and the late phase of the CPT. The dCA was examined using a thigh cuff occlusion and release technique. The MCA and PCA blood velocities were unchanged at CPT compared with the control conditions despite an elevation in the ABP. The dCA in both the MCA and PCA remained unaltered at CPT. These findings suggest that CPT-induced elevations in the ABP and SNA did not cause changes in the CBF regulation in the posterior circulation compared with the anterior circulation.

## Introduction

Interestingly, hypertensive disease-induced primary intracerebral hemorrhage occurs mainly at the small penetrating blood vessels in the posterior cerebral circulation rather than the anterior cerebral circulation [1]. Hypertension is a risk factor for cerebrovascular diseases [2, 3]; therefore, these findings suggest that the posterior cerebral vasculature may not be able to properly regulate an elevation in blood pressure compared with the anterior cerebral vasculature. Recent studies [4–8] have reported that the responses of the posterior cerebral blood flow (CBF) in several physiological conditions (e.g., orthostatic stress, hypoxia, dynamic resistance exercise, heat stress, etc.) are different from those of the anterior CBF. Indeed, it has been reported that the dynamic cerebral autoregulation (dCA) or cerebrovascular carbon dioxide (CO_2_) reactivity is lower in the posterior cerebral circulation than that of anterior cerebral circulation [9, 10]. It is well established that these cerebral regulatory mechanisms keep the CBF relatively constant despite changes in cerebral perfusion pressure (CPP), which is critical in preventing cerebral ischemia or hemorrhage [11]. Although it can be expected that this heterogeneous CBF response may be due to different physiological roles, the mechanism of this heterogeneous CBF remains unknown.

On the other hand, some previous studies [12–14] reported that sympathetic blockade (prazosin or trimethaphan) impairs dCA, suggesting that autonomic neural control, i.e., sympathetic nerve activity (SNA), of the cerebral circulation likely plays an important role in the dynamic CBF regulation. The SNA also exerts a greater influence on cerebral circulation in hypertension than in the normotensive condition [15]. Thus, sympathoexcitation accompanied by hypertension may improve dCA and prevent over-perfusion. Importantly, there are anatomical differences between the anterior and posterior cerebral circulations, including regional heterogeneity in the sympathetic innervation of intracranial arterioles [16]. The posterior cerebral circulation may have less sympathetic innervation than the anterior cerebral circulation [16]. Therefore, an effect of sympathoexcitation on dynamic CBF regulation may be less in the posterior cerebral circulation compared with anterior cerebral circulation. Hypertension-induced sympathoexcitation may emphasize the difference between anterior and posterior CBF regulation, and this phenomenon may be associated with hypertensive disease-induced primary intracerebral hemorrhage occurred in mainly at the posterior cerebral circulation. However, an effect of sympathoexcitation on posterior CBF regulation has not been identified.

Against this background, we hypothesized that the dCA or cerebral vasculature tone of the posterior CBF is lower than that of the anterior CBF during an elevation in the arterial blood pressure (ABP) accompanied with sympathoexcitation. To test this hypothesis, the CBF velocity as an index of CBF, cerebral vascular tone, and the dCA in the middle cerebral artery (MCA) and the posterior cerebral artery (PCA) were measured before and during a cold pressor test (CPT). The CPT is widely used as a physiological tool to evoke temporary increases in the ABP with an elevation in the SNA.

## Methods

### Ethical approval

The protocol was approved by the Institutional Review Board at Toyo University (TU-2017-004) and each subject provided written informed consent prior to participating in the study. The study was performed in accordance with the principles of the Declaration of Helsinki.

### Subjects

A total of 11 young men (mean ± SD, age 22 ± 1 years, height 175 ± 5 cm, weight 65 ± 8 kg) participated in this study. They did not have any cerebrovascular or cardiovascular disease and were not taking any medications at the time of enrollment. Prior to each experimental session, the participants were required to abstain from caffeine for 12 h and strenuous exercise and alcohol for 24 h. The experiment was performed at least 3 h after a light meal.

### Experimental protocol

**Fig. 1:**
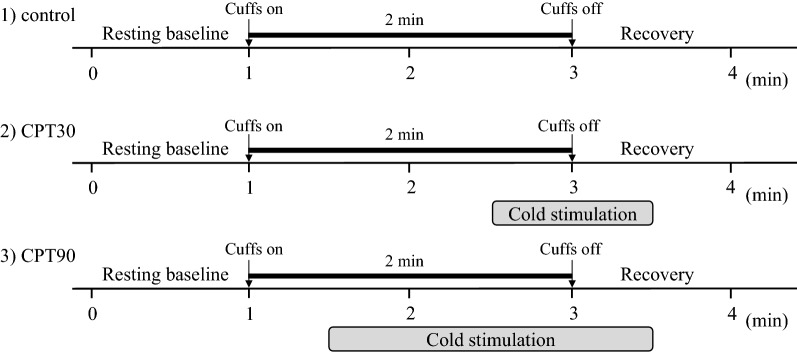
Overview of the experimental protocol

Following instrumentation, the subjects were asked to rest in the supine position on a bed for at least 15 min before the commencement of the protocol. Each subject had the thigh cuff occlusion-release protocol at three different conditions: control, early phase of the CPT (cuff release at the 30th s of CPT, CPT30), and the late phase of the CPT (cuff release at the 90th s of CPT, CPT 90, Fig. 1). Previous studies [17] suggest that the cerebrovascular response may be modified by cold stimulation-induced pain sensation as well as elevations in ABP and SNA during a CPT. In the present study, to identify the cerebrovascular response isolated from pain sensation, the dCA and critical closing pressure (CrCP) were assessed at two different time phases (CPT30 and CPT90). This is because the pain sensation is not changed during a CPT [18], whereas the ABP and SNA increase gradually and reach their peaks at 60–90 s after cold stimulation [19]. Thus, the different measurement parameters between CPT30 and CPT90 may be due to elevations in the ABP and SNA and not pain sensation.

Each subject immersed their left hand into 1–2 °C water starts at the 90th or 30th s after cuff inflation during CPT30 or CPT90, respectively, and continued to immerse until 30 s after cuff deflation. All trials were randomized and separated by a minimum of 15 min for the hemodynamics to return to the baseline. The thigh cuff occlusion-release protocol uses acute hypotension to identify the dCA [20]. Each test was performed twice; thus, each subject performed six total trials of the thigh cuff occlusion-release protocol. The thigh cuff occlusion-release technique consists of a resting baseline of 1-min, 2-min inflation of thigh cuffs (> 180 mmHg), and a 30-s deflation period. Throughout the experimental protocol, the subjects were instructed to adjust their respiratory rate (RR) according to the sound of a metronome (15 breaths/min) to maintain the same end-tidal partial pressure of carbon dioxide (P_ET_CO_2_) level [21].

### Experimental measurements

The heart rate (HR) was measured using a lead II electrocardiogram (bedside monitor, BMS‐3400; Nihon Kohden, Japan). The beat-to-beat arterial blood pressure was monitored continuously using finger photoplethysmography (Finapres Medical Systems, Amsterdam, The Netherlands) to determine the systolic blood pressure (SBP), diastolic blood pressure (DBP), and mean arterial pressure (MAP). The stroke volume (SV) was determined from the BP waveform using the Modelflow software program, which incorporates the sex, age, height, and weight of the subject (Beat Scope1.1; Finapres Medical Systems BV). The cardiac output (CO) was calculated by the SV multiplied by the HR. The P_ET_CO_2_, minute ventilation (V_E_), and RR were sampled from a leak-free mask and measured with a gas analyzer (AE-310S; Minato Medical Science Co., Osaka, Japan). Cerebral blood velocity in the right MCA (MCAv) and left PCA (PCAv) were measured as an index of anterior and posterior CBF, respectively, using a 2-MHz pulsed transcranial Doppler (TCD) probe (DWL Doppler Box-X; Compumedics, Germany). The TCD probe was fixed and held in place using a headband.

Participants were asked to indicate their pain level immediately after a CPT on a 10 cm visual analog scale (VAS). The VAS for pain intensity was anchored with “no pain” at one end and “severe pain” at the other end [17].

### Data analysis

The beat-to-beat MAP, MCAv, and PCAv were obtained from each waveform. The cerebrovascular conductance index of the MCA (MCA CVCi) or PCA (PCA CVCi) was calculated by dividing the MCAv or PCAv, respectively, by the MAP. The hemodynamic data were averaged using 30 s data points before cuff deflation and 30 s data point before end of cuff deflation at each condition. The dCA was the average of values in two trials at each condition.

As previously reported, the CrCP of the cerebral circulation was estimated as the index of cerebral vascular tone [22]. Pairs of systolic and diastolic values of MCAv or PCAv and ABP were used to determine pressure–flow velocity relationships, i.e., CrCP [23–26]. The ABP axis intercept of the extrapolated regression line between 30 s of consecutive pairs of systolic and diastolic values of ABP (*x*-axis, mmHg) and MCAv or PCAv (*y*-axis, cm/s) waveforms determines the CrCP [22, 27].

We have calculated dCA in both MCA and PCA at each condition according as previous study [20]. The prerelease values of CBF (proportional to velocity) and MAP were defined by their means during the 4 s before thigh cuff release. The relative changes to prerelease value in the MAP, MCAv or PCAv, and MCA CVCi or PCA CVCi during thigh cuff release value were calculated (value/baseline value; normalized units relative to control prerelease values). A slope of the relationship between relative changes to prerelease in the MAP and MCA CVCi or PCA CVCi from 1.0 to 3.5 s after the cuff release was computed as the rate of regulation (RoR), which was used as an index of dCA [20]:

$${\text{RoR}}\;(/{\text{s}}) = \left( {{{\Delta {\text{MCA}}\;{\text{CVCi}}\;{\text{or}}\;\Delta {\text{PCA}}\;{\text{CVCi}}} \mathord{\left/ {\vphantom {{\Delta {\text{MCA}}\;{\text{CVCi}}\;{\text{or}}\;\Delta {\text{PCA}}\;{\text{CVCi}}} {\Delta T}}} \right. \kern-\nulldelimiterspace} {\Delta T}}} \right)\Delta {\text{MAP}} ,$$

where (ΔMCA CVCi or ΔPCA CVCi/ Δ*T*) is the slope of the linear regression between MCA CVCi or PCA CVCi and time (*T*), and ΔMAP, the magnitude of the step, was calculated by subtracting control MAP from the averaged MAP during the interval from 1.0 to 3.5 s [20].

### Statistical analysis

All data are expressed as mean ± SD. One-way analysis of variance (ANOVA) with repeated measures was used to compare for hemodynamics each condition (control, CPT30, and CPT90). Two-way ANOVA with repeated measures (3 conditions; control, CPT30, and CPT90 × 2 arteries; MCA or PCA) was performed with Bonferroni’s post hoc test (SPSS 24, IBM, Tokyo, Japan) where appropriate. Effect sizes for ANOVA are reported as partial eta squared (*η*_*p*_^2^). *P* values of < 0.05 were regarded as statistically significant.

## Results

Any differences in hemodynamics during the resting baseline between the different conditions were not observed (*P* > 0.238), suggesting that the 15-min interval between the test conditions was enough for the recovery of hemodynamics to the baseline value.

There was no significant difference in the subjective pain sensation between the CPT30 (6.94 ± 2.24) and CPT90 (7.45 ± 2.13) (*P* = 0.347). At the CPT30, DBP, SV, and CO were unchanged (*P* > 0.234), but the HR, SBP, and MAP increased compared with the control conditions (*P* < 0.025) (Table 1). In addition, at CPT90, SBP, and DBP, the MAP increased further from the CPT30 (*P* < 0.008), but the HR did not change (*P* = 1.00). Because the subjects maintained their voluntary RR during the experiment (*P* = 0.883), *V*_E_ and P_ET_CO_2_ were unchanged compared with the control throughout the CPT (*P* > 0.065).

**Table 1:** Hemodynamic parameters at control, CPT30, and CPT90

	Control	CPT30	CPT90
HR (bpm)	70.2 ± 17.7	81.5 ± 17.1*	81.0 ± 15.5*
SBP (mmHg)	129.1 ± 11.2	139.9 ± 11.1*	152.1 ± 15.8*^†^
DBP (mmHg)	74.0 ± 8.3	78.6 ± 8.3	87.8 ± 8.4*^†^
MAP (mmHg)	92.4 ± 8.4	99.0 ± 8.2*	109.2 ± 10.2*^†^
SV (mL)	90.8 ± 16.9	94.0 ± 12.4	92.0 ± 11.6
CO (L/min)	6.3 ± 1.8	7.6 ± 1.8	7.4 ± 1.7
RR (breath/min)	14.8 ± 0.7	14.8 ± 0.7	14.8 ± 0.6
*V*_E_ (L/min)	8.3 ± 1.6	8.7 ± 1.4	9.9 ± 2.5
P_ET_CO_2_ (mmHg)	37.5 ± 2.7	37.2 ± 1.9	36.4 ± 2.5
MCAv (cm/s)	58.8 ± 6.1	60.4 ± 10.3	59.8 ± 12.7
MCA CVCi (cm/s/mmHg)	0.64 ± 0.05	0.61 ± 0.09	0.55 ± 0.11
MCA CrCP (mmHg)	16.7 ± 13.5	10.9 ± 13.2	6.0 ± 16.6*
PCAv (cm/s)	33.3 ± 7.3	33.4 ± 6.7	33.1 ± 8.2
PCA CVCi (cm/s/mmHg)	0.36 ± 0.07	0.34 ± 0.07	0.30 ± 0.07*
PCA CrCP (mmHg)	13.6 ± 15.9	4.6 ± 14.4*	4.1 ± 19.9

Data are presented as means ± SD

*HR* heart rate, *SBP* systolic blood pressure, *DBP* diastolic blood pressure, *MAP* mean arterial pressure, *SV* stroke volume, *CO* cardiac output, *RR* respiratory rate, *V*_*E*_ ventilation, *P*_*ET*_*CO*_*2*_ end-tidal partial pressure of carbon dioxide, *MCAv* middle cerebral artery mean blood velocity, *MCA CVCi* middle cerebral artery cerebral vascular conductance index, *MCA CrCP* middle cerebral artery critical closing pressure, *PCAv* posterior cerebral artery mean blood velocity, *PCA CVCi* posterior cerebral artery cerebral vascular conductance index, *PCA CrCP* posterior cerebral artery critical closing pressure

^* ^*P* < 0.05 different from control, ^†^*P* < 0.05 different from CPT30

**Fig. 2:**
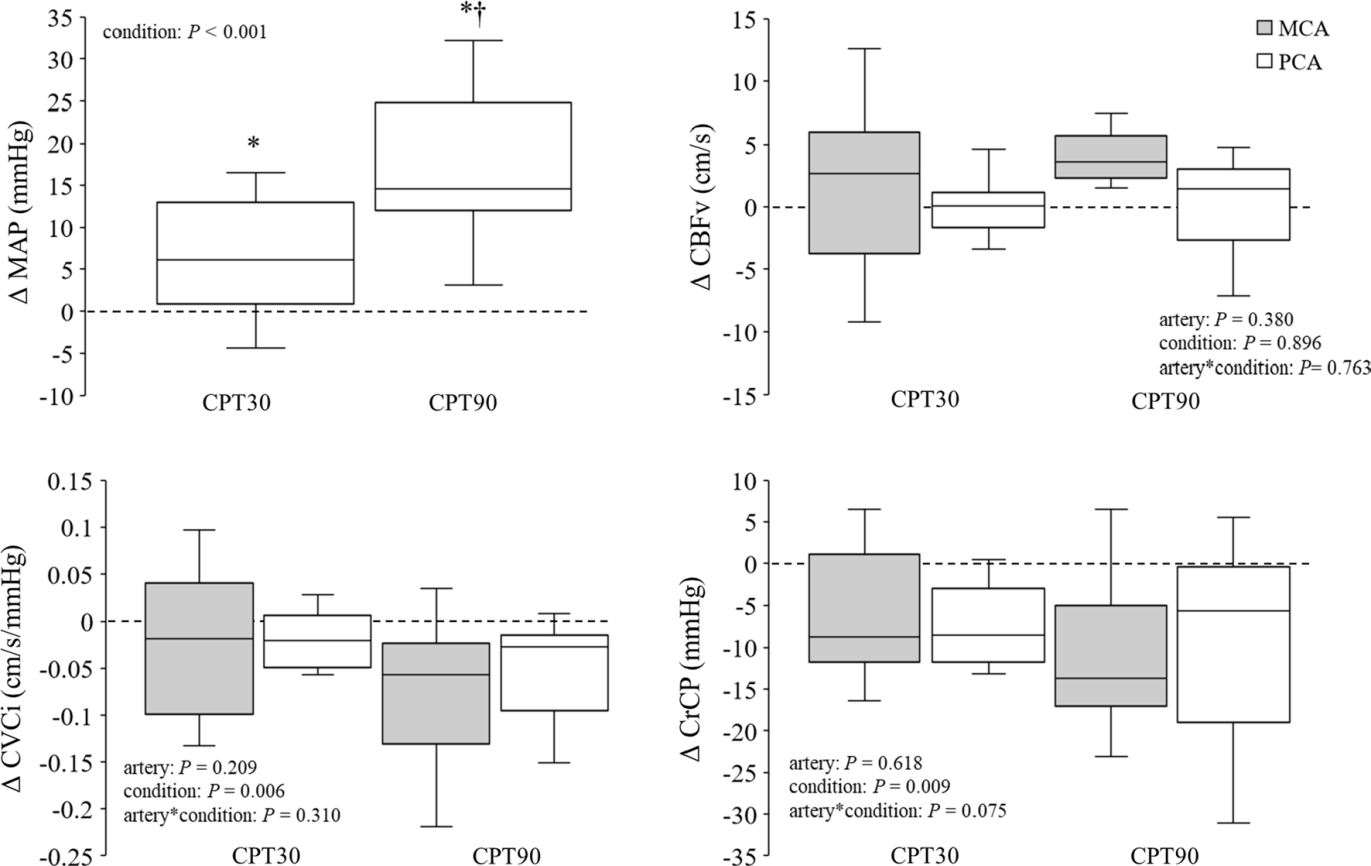
Change in mean arterial blood pressure (MAP), cerebral blood flow velocity (CBFv), cerebral vascular conductance index (CVCi), and critical closing pressure (CrCP) from the control during CPT30 and CPT90 (*n* = 11). *P* value represents repeated two-way ANOVA results. ^*^P < 0.05 different from control, ^†^*P* < 0.05 different from CPT30. Data are presented as means ± SD

Both the MCAv and PCAv were unchanged at the CPT30 and CPT90 compared with control condition (MCA; *P* = 0.846, PCA; *P* = 0.958, Table1). The CVCi was decreased in both the MCA and PCA (MCA; *P* = 0.053, PCA; *P* = 0.022, Table1), suggesting that CPT causes cerebral vasoconstriction. However, there was no significant difference in changes in the CVCi between MCA and PCA (*P* = 0.310, *η*_*p*_^2^ = 0.111; Fig. 2). MCA CrCP and PCA CrCP were lower at the CPT90 and CPT30 (*P* = 0.012 and *P* = 0.019, respectively) compared with the control (Table 1). Although the change in the MCA CrCP and PCA CrCP reduced during CPT, the change in the MCA CrCP from the control conditions was not different from that of the PCA CrCP (*P* = 0.075, *η*_*p*_^2^ = 0.229; Fig. 2).

**Fig. 3:**
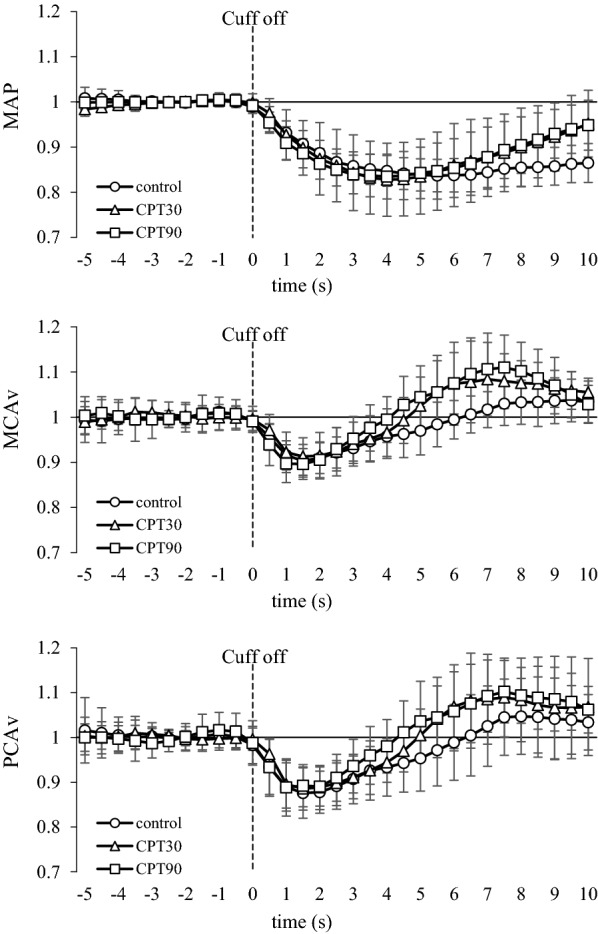
Normalized averaged data of mean arterial pressure (MAP), blood flow velocity of middle (MCA), and posterior cerebral arteries (PCA) to thigh cuff release during control, CPT30, and CPT90 (*n* = 11). Thigh cuff deflation occurred at time 0. All data are shown in normalized units relative to pre-deflation values obtained during − 4 to 0 s

**Fig. 4:**
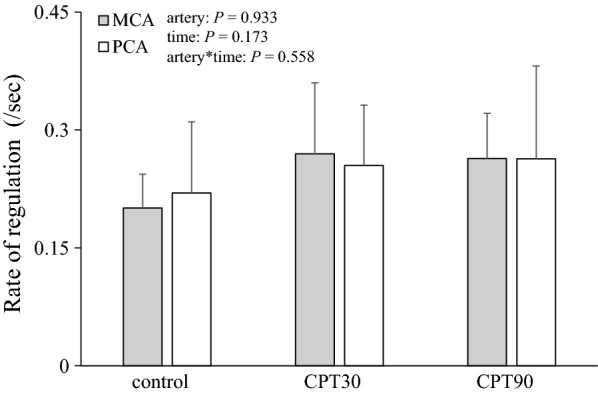
Rate of regulation (RoR) as an index of dynamic cerebral autoregulation at control, CPT30, and CPT90 (*n* = 11). *P* value represents repeated two-way ANOVA results. Data are presented as means ± SD

The release of the thigh cuffs elicited an acute decrease in the ABP at all the test conditions (Fig. 3). Changes in the MAP at both CPT30 and CPT90 were similar to the control conditions (*P* = 0.601). The nadir of the MCAv and PCAv responses to cuff release was not different among the three conditions (*P* = 0.579, *η*_*p*_^2^ = 0.053; Fig. 3), and there was no difference in the RoR as an index of dCA between conditions or arteries (*P* = 0.558, *η*_*p*_^2^ = 0.057; Fig. 4).

## Discussion

The present study examines whether CBF regulation in the posterior cerebral circulation is different from the anterior cerebral circulation during a CPT. Both the MCAv and PCAv were unchanged at the CPT30 and CPT90 from the baseline, despite an elevation in the ABP. In addition, the dCA in both MCA and PCA remained unaltered at CPT30 and CPT90. Interestingly, the MCA CrCP and PCA CrCP, an index of cerebral vascular tone, decreased during the CPT. However, there was no difference in the CrCP between the MCA and PCA. In contrast to our hypothesis, these findings indicate that CPT did not cause any difference in CBF regulation between the posterior and anterior cerebral circulations. These findings indicate that both the anterior and posterior cerebral vasculature were well-regulated (i.e., no over-perfusion occurs) via the dCA despite CPT-induced high blood pressure accompanied with a high SNA.

Unexpectedly, the posterior CBF response to the CPT was not different from that of the anterior CBF (Table 1 and Fig. 2). In addition, both MCAv and PCAv were unchanged during the CPT (Table 1 and Fig. 2), suggesting that the posterior and anterior cerebral circulations were well-regulated despite an elevation in the ABP. Generally, blood flow is determined by the balance between perfusion pressure and vasomotion. However, the cerebral circulation has a dCA, a specific physiological mechanism that maintains adequate cerebral perfusion against a variation in the ABP. Indeed, impairments in the dCA are associated with cerebrovascular diseases [28, 29]. Therefore, the present study identified the dCA and CrCP as indices of cerebral vascular tone during the CPT to address the mechanisms of CBF response to the CPT. As with the CBF response, the response of the dCA and CrCP to the CPT in the posterior cerebral circulation was not different from that of the anterior cerebral circulation. Based on this, the lack of a difference in CBF between the posterior and anterior cerebral circulation was likely due to the response of the dCA and CrCP to the CPT.

The CrCP is a well-established index of cerebral vascular tone, influenced by changes in metabolism (e.g., CO_2_ and neural activities), transmural pressure (e.g., intracranial pressure), and the SNA [13, 22, 30]. Moreover, previous studies have suggested that an increase in the CrCP might protect the blood–brain barrier from the over-perfusion induced by exercise-induced hypertension [23, 24]. It has been well-established that there is regional heterogeneity in the sympathetic innervation of the intracranial artery [15]. Therefore, we expected that the CPT will lead to differences in the CrCP and different CBF between the MCA and PCA because increases in the SNA and BP enhance the CrCP [13, 22]. Unexpectedly, the MCA CrCP or PCA CrCP decreased rather than increase at the CPT30 (*P* = 0.019, Fig. 2) and CPT90 (*P* = 0.012, Fig. 2) compared with the control conditions despite an elevation in the ABP. In addition, there was no difference in the change in the CrCP and CBF from the control conditions between the MCA and PCA (*P* = 0.075; Fig. 2). Therefore, the decrease in the CrCP is not consistent with the concept that the cerebral vascular response protects the blood–brain barrier from over-perfusion. Although the CBF response to CPT may be associated with no difference in the response of the CrCP to the CPT between the anterior and posterior cerebral circulation, it is unclear how CPT-induced decreases in the CrCP led to no changes in the CBF with an elevation in the ABP. The CrCP is an index of changes in smaller arterioles, which are more responsive to regional brain metabolic demands [30, 31]. Thus, the reduction in the CrCP may be associated with neural activity-induced metabolic changes at the regional level. Neuroimaging studies have shown that the neural activity of the brainstem is increased at the initial phase of a CPT, whereas high-order regions are activated at the later phase [32, 33]. Therefore, CPT-induced decreases in the CrCP may be affected by neural activation in the brain area supported by the MCA and PCA. However, the CVCi decreased in both the MCA and PCA, suggesting that CPT causes cerebral vasoconstriction. The physiological mechanism responsible for the decrease in CrCP during CPT despite an increase in vascular resistance in the cerebral artery remains unclear.

In the present study, the RoR as an index of the dCA in the MCA and PCA was unchanged during CPT. In contrast to these results, Hilz et al. [34] reported that CPT impaired the dCA in the MCA. One possible reason for this inconsistent result is that the foot was stimulated rather than the hand for the CPT. Although there was no difference in the pressor response during CPT between the hand and foot, the endocrine response and subjective pain sensation during cold stimulation of the foot were larger compared with that of the hand [35]. Because it has been reported that pain intensity differences could modify the CBF response [36], different pain sensation due to different stimulation methodology may account for be the inconsistent result.

In contrast to our hypothesis, the RoR as an index of the dCA in the PCA was unchanged at CPT30 and CPT90, but was not different from that of the MCA (*P* = 0.558; Fig. 3). It has been reported that the dCA is lower in the posterior cerebral circulation than in the anterior cerebral circulation under normal physiological conditions [37, 38], but these findings are inconsistent [39, 40]. Alternatively, few studies have compared the anterior and posterior dCA during physiological stimulation. Interestingly, though there is no difference in the dCA between the anterior and posterior cerebral circulation under the supine position, orthostatic stress evokes regional differences in the dCA [10]. This heterogeneous dCA may be partially related to regional heterogeneity in the sympathetic innervation of intracranial arterioles. Indeed, the posterior cerebral circulation may have a less sympathetic innervation than the anterior cerebral circulation [16]. This finding indicates that if the SNA partly contributes to the cerebral vasculature, CPT-induced sympathoexcitation influences are smaller in the posterior circulation than in the anterior circulation and, consequently, cause less cerebral vasoconstriction and less augmented dCA in the posterior circulation. This concept may support the finding that hypertensive disease-induced primary intracerebral hemorrhage occurs mainly in the posterior cerebral circulation rather than the anterior cerebral circulation [1] because the vascular response has a weaker capacity to protect the blood–brain barrier against cerebral over-perfusion during an acute elevation in the ABP. However, in the present study, both the MCAv and PCAv were unchanged during CPT. Moreover, in contrast to our hypothesis, the posterior cerebral circulation was well-regulated despite an elevation in the ABP and SNA. An intact dCA during CPT in the posterior cerebral circulation may be associated with an adequate posterior CBF that is preserved during acute elevations in the ABP and SNA. In addition, these results suggest that the effects of different SNA between the anterior and posterior cerebral circulation during CPT on the dCA may be minimal.

## Limitations

Some potential limitations of the present study should be considered. First, the TCD-determined MCAv and PCAv can be used as indices of the anterior and posterior CBF, with the assumption of a constant diameter of the insonated artery. In this regard, several studies have reported no change in the MCA diameter in response to physiological stimulations such as orthostatic stress [41, 42]. In contrast, a recent report demonstrated that dynamic handgrip exercise response caused significant changes in the MCA diameter [43]. However, if sympathetic activation during CPT elicits a decrease in the MCA or PCA diameter, measuring changes in the MCAv or PCAv would overestimate the anterior or posterior CBF because a reduction in diameter would increase blood velocity. Thus, because there is no change in the MCAv and PCAv during CPT, it can be inferred that changes in the MCAv and PCAv likely reflect changes in blood flow. Second, a few previous studies have suggested that the dCA is relatively more effective at protecting the brain against transient hypertension than hypotension [44, 45]. Therefore, our findings may not reflect the RoR using transient hypertensive stimuli. Third, we did not examine the effect of CPT without cuff inflation on hemodynamics response to reduce the burden of subjects in the present study. However, there was no significant difference in MAP, MCAv, and PCAv between the resting baseline and cuff inflation (MAP; *P* = 0.179, MCA; *P* = 0.114, PCA; *P* = 0.201). Therefore, the effect of cuff inflation on hemodynamics response to CPT may be minimal. Finally, the participants in the present study were young healthy subjects. Therefore, the results may differ in older aged individuals with a greater risk of hypertension or stroke. Indeed, a previous study reported that the CBF response to cold stimulation is different between older and young individuals [46]. Thus, a dedicated study on the effect of aging in CBF regulation during CPT in humans is required to address this issue.

## Conclusion

Contrary to our hypothesis, there was no difference in CBF and its regulation between the anterior and posterior cerebral circulation during an elevation in ABP and SNA. This finding suggests that CPT-induced acute elevations in blood pressure and SNA did not affect the regulation of posterior cerebral vasculature, and the posterior CBF and the anterior cerebral circulation was well-regulated. However, the effects of a chronic elevation in ABP (hypertension) on the posterior cerebral circulation may be different from that of an acute elevation in ABP.

## Data Availability

All relevant data are within the paper.

## Abbreviations

ABP: arterial blood pressure
CBF: cerebral blood flow
CO_2_: carbon dioxide
CO: cardiac output
CPP: cerebral perfusion pressure
CPT: cold pressor test
CrCP: critical closing pressure
CVCi: cerebrovascular conductance index
DBP: diastolic blood pressure
dCA: dynamic cerebral autoregulation
HR: heart rate
MAP: mean arterial pressure
MCA: middle cerebral artery
PCA: posterior cerebral artery
P_ET_CO_2_: end-tidal partial pressure of carbon dioxide
RoR: rate of regulation
RR: respiratory rate
SBP: systolic blood pressure
SNA: sympathetic nervous activity
SV: stroke volume
VAS: visual analog scale
VE: minute ventilation
